# Ets-1 regulates intracellular glutathione levels: key target for resistant ovarian cancer

**DOI:** 10.1186/1476-4598-12-138

**Published:** 2013-11-15

**Authors:** Meghan L Verschoor, Gurmit Singh

**Affiliations:** 1 Department of Pathology and Molecular Medicine, McMaster University, 1280 Main Street W, Hamilton, Ontario 12943, Canada

**Keywords:** Ets-1, Glutathione, Ovarian cancer, Oxidative stress, Drug resistance

## Abstract

**Background:**

Ovarian cancer is characterized by high rates of metastasis and therapeutic resistance. Many chemotherapeutic agents rely on the induction of oxidative stress to cause cancer cell death, thus targeting redox regulation is a promising strategy to overcome drug resistance.

**Methods:**

We have used a tetracycline-inducible Ets-1 overexpression model derived from 2008 ovarian cancer cells in the present study. To examine the role of Ets-1 in glutathione regulation we have measured intracellular reactive oxygen species and glutathione levels, as well as glutathione peroxidase enzyme activity. Glutathione synthesis was limited using transsulfuration or Sx_c_^-^ pathway blocking agents, and glutamate release was measured to confirm Sx_c_^-^ blockade. Cell viability following drug treatment was assessed via crystal violet assay. Oxidative stress was induced through glucose oxidase treatment, which produces hydrogen peroxide by glucose oxidation. The protein expressions of redox-related factors were measured through western blotting.

**Results:**

Overexpression of Ets-1 was associated with decreased intracellular ROS, concomitantly with increased intracellular GSH, GPX antioxidant activity, and Sx_c_^-^ transporter activity. Under basal conditions, inhibition of the transsulfuration pathway resulted in decreased GSH levels and GPX activity in all cell lines, whereas inhibition of Sx_c_^-^ by sulfasalazine decreased GPX activity in Ets-1-expressing cells only. However, under oxidative stress the intracellular GSH levels decreased significantly in correlation with increased Ets-1 expression following sulfasalazine treatment.

**Conclusions:**

In this study we have identified a role for proto-oncogene Ets-1 in the regulation of intracellular glutathione levels, and examined the effects of the anti-inflammatory drug sulfasalazine on glutathione depletion using an ovarian cancer cell model. The findings from this study show that Ets-1 mediates enhanced Sx_c_^-^ activity to increase glutathione levels under oxidative stress, suggesting that Ets-1 could be a promising putative target to enhance conventional therapeutic strategies.

## Background

Ovarian cancer, much like several types of human cancer, remains extremely challenging to treat in advanced stages despite prolific therapeutic advances. Patients with ovarian cancer generally have a poor prognosis, as most women are not diagnosed until the disease has reached advanced stages. Surgical intervention is the most successful treatment, however, due to the close proximity of other peritoneal structures to the ovary, recurrence of the disease within nearby organs is very common [1]. As recurrent ovarian cancer remains a largely incurable state of disease, it is vitally important to examine the characteristic differences between early and late stage ovarian cancers, and mechanisms of therapeutic resistance. Cisplatin and other platinum drugs are the most common first round chemotherapeutic agents used to treat ovarian cancer, where most patients who receive treatment experience platinum-sensitive recurrence, typically followed by the eventual development of platinum drug resistance [2].

The specific mechanisms involved in the development of therapeutic resistance are not fully understood, however it is well-recognized to be a multi-factorial process involving alterations in drug metabolism and transport, DNA damage signaling and repair, apoptosis and survival signaling, as well as off-target changes that counteract the lethal effects of the chemotherapy [2]. Recently, cancer stem cells have been revealed as a key cell type involved in the recurrence and drug resistance of ovarian cancer [2-6]. Due to the heterogeneous nature of ovarian cancer tumors, it is thought that primary tumours are composed of drug sensitive cells, resistant cells, and cancer initiating stem cells. In this theory, the bulk of primary tumours are composed of sensitive cells that succumb to initial chemotherapy treatment, however the population of cancer stem cells that are drug resistant repopulate the tumour resulting in drug resistance upon recurrence.

Importantly, drug resistance in ovarian cancer is often characterized by the acquisition of genetic modifications in key signaling pathways such as p53 and Akt [7]. Our research group has previously established that the transcription factor v-ets erythroblastosis virus E26 oncogene homolog 1 (Ets-1) is associated with chemotherapeutic resistance in ovarian cancer cells, whereby increased expression of Ets-1 results in decreased sensitivity to cisplatin [8]. More recently, we have established that Ets-1 regulates energy metabolism in ovarian cancer cells by enhancing glycolytic dependence, and also extended these findings to a breast cancer model [9,10]. The increased glycolytic utilization observed was paired with increases in the expression of several pentose phosphate pathway genes. This pathway is one of the major sources of NADPH that is required for glutathione reduction, which is an important factor in therapeutic resistance. Therefore, because Ets-1 overexpression leads to increased glycolytic flux, it is possible that Ets-1 is also involved in the regulation of cellular redox state since these processes are intrinsically linked.

The balance between cell death and proliferation is tightly controlled in healthy cells, whereas in cancer cells this equilibrium is shifted towards a proliferative state with enhanced survival. Imbalances in cellular redox state are frequently observed in cancer cells, and contribute significantly to cancer progression and apoptotic resistance. Reduced glutathione (GSH) is the most abundant non-protein thiol in mammalian cells, and tightly regulates redox state through its antioxidant and reducing activities. Additionally, GSH is involved in the control of cell cycle regulation, proliferation, apoptosis, and therapeutic resistance in cancer cells [11-14]. Many chemotherapeutic agents and radiation treatments depend on the alteration of redox state through the induction of oxidative stress via reactive oxygen species (ROS) generation to kill cancer cells. Changes in GSH levels affect mitochondrial pore permeability, where depletion of GSH leads to the release of cytochrome c and cell death, thus rendering GSH an attractive target for cancer therapy that could ameliorate the success of conventional therapies [15]. Interestingly, glutathione levels are higher in ovarian tumours than in healthy ovarian tissue, and increase in patients who are non-responsive to therapeutic intervention [16,17].

In this study, we reveal that Ets-1 elevates cellular glutathione levels in ovarian cancer cells, which could account for the therapeutic resistance associated with this transcription factor. We have shown that ovarian cancer cells that overexpress Ets-1 display decreased intracellular ROS, with increased intracellular GSH and glutathione peroxidase (GPX) antioxidant activity. The availability of intracellular cysteine is rate-limiting for GSH synthesis, and is determined by the activities of the transsulfuration pathway and the membrane antiporter System x_c_^-^ (Sx_c_^-^), which imports cystine in exchange for glutamate release. We have used inhibitors of each of these systems to determine whether Ets-1 plays a role in the regulation of either pathway, thus explaining the changes in GSH that we have observed. Our results show that Ets-1 mediates enhanced Sx_c_^-^ activity to increase glutathione recycling in ovarian cancer cells, and that this effect was enhanced during oxidative stress. To our knowledge, this is the first study to report that Ets-1 regulates glutathione levels in cancer cells, suggesting that Ets-1 could be a promising putative target to increase the effectiveness of therapeutic strategies dependent on GSH depletion.

## Results

### Ets-1 decreases intracellular ROS, while increasing intracellular GSH and GPX activity

**Figure 1:**
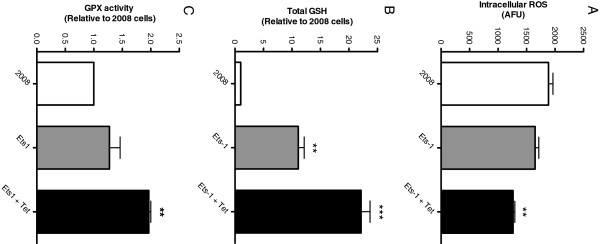
**Ets-1 decreases intracellular ROS, while increasing intracellular GSH and GPX activity. A)** Basal intracellular ROS levels were measured in 2008, 2008-Ets1 (Ets-1), and tetracycline-induced 2008-Ets1 (Ets-1 + Tet) cells using CM_2_-H_2_DCFDA. There is a trend for decreased ROS levels in correlation with increased Ets-1 expression (n = 3). **B)** Total intracellular amounts of GSH were measured using an enzymatic recycling method as described by Rahman *et al.* (12). Metabolite extracts from 2008, Ets-1 and induced Ets-1 cells contained increased GSH levels concomitantly with increased Ets-1 expression (n = 4). **C)** Glutathione peroxidase activity also displayed a trend towards increased activity in association with higher levels of Ets-1 expression (n = 4).

To examine the role of Ets-1 in the regulation of cellular antioxidant capacity, we measured intracellular ROS levels, total cellular GSH, and GPX enzyme activity. Intracellular ROS was measured using CM_2_-H_2_DCFDA in 2008, 2008-Ets1, and induced 2008-Ets1 cells (Figure 1A). The amount of ROS present in induced 2008-Ets1 cells (1264.4 AFU) was significantly less than their parental 2008 cells (1885.5 AFU), while non-induced 2008-Ets1 cells were not significantly different. Quantitative total GSH was measured via colourimetric assay under basal conditions, and both 2008-Ets1 and induced 2008-Ets1 cells showed increased amounts of total GSH relative to 2008 cells (11.11- and 22.14-fold respectively) (Figure 1B). GPX enzyme activity was assessed by measure of the oxidation of NADPH, and induced 2008-Ets1 cells were found to have a higher GPX activity rate (1.96-fold) than 2008 cells (Figure 1C).

### Ets-1 increases System x_c_^-^ expression and activity

**Figure 2:**
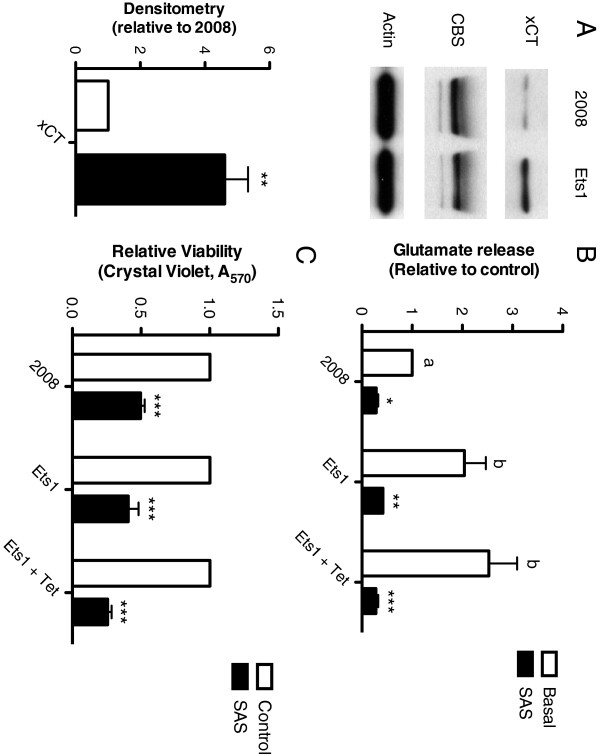
**Ets-1 increases System x**_**c**_^**- **^**expression and activity. A)** The protein expression of the catalytic subunit of Sx_c_^-^, xCT, and the transsulfuration pathway enzyme CBS were compared in 2008 and induced 2008-Ets1 cells via Western Blot. Though the expression of CBS was not different, xCT protein levels were increased in response to Ets-1 overexpression (n = 3). **B)** Glutamate release was measured by AMPLEX red® fluorescence in 2008 and 2008-Ets1 cells under basal or SAS-treated conditions. Cells that overexpress Ets-1 release more glutamate into the culture medium, and SAS was an effective inhibitor of glutamate release (n = 3). **C)** Cell viability was measured via Crystal Violet assay following SAS treatment, which was found to decrease viability in all cell lines (n=3).

The protein expression of xCT, the subunit of Sx_c_^-^ responsible for transporter activity of the antiporter, and of CBS, which catalyzes the first step of the transsulfuration pathway, were examined in 2008 and induced 2008-Ets1 cells (Figure 2A). The expression of xCT was found to be 4.6-fold higher in induced 2008-Ets1 cells compared to parental 2008 cells via densitometry analysis. No significant difference in the expression of CBS was found between 2008 and induced 2008-Ets1 cells. The amount of glutamate released into the culture medium was measured, and used to test the effectiveness of SAS in blocking Sx_c_^-^ transporter activity. Both non-induced and induced 2008-Ets1 cells released more glutamate into the culture environment than parental 2008 cells (2.04-fold and 2.53-fold respectively)(Figure 2B). Treatment with SAS, an inhibitor of Sx_c_^-^, resulted in a significant reduction of extracellular glutamate for all cell lines. Additionally, SAS treatment significantly decreased cell viability in all cell types of our model system (Figure 2C), a finding that is in accordance with previously published work by Lo *et al.*[18].

### The transsulfuration pathway is a major GSH source in ovarian cancer cells

**Figure 3:**
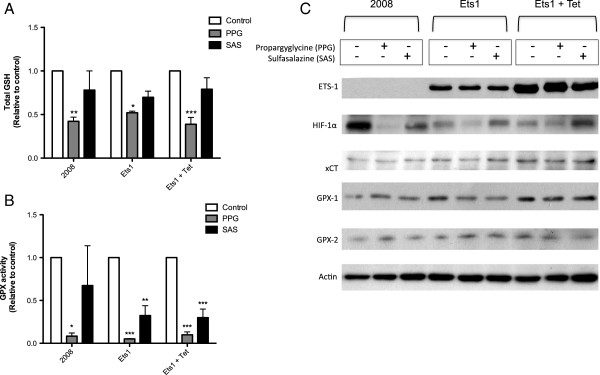
**The transsulfuration pathway is a major GSH source in normoxic ovarian cancer cells.** 2008, 2008-Ets1 and induced 2008-Ets1 ovarian cancer cells were treated with PPG or SAS for 24 hrs, and the redox capacity was examined. **A)** Quantitative total GSH levels were significantly decreased in response to PPG treatment in all cell lines, while SAS did not significantly affect GSH levels (n = 3). **B)** Like total GSH levels, enzyme activity of GPX was significantly decreased by PPG-mediated blockade of the transsulfuration pathway. SAS treatment resulted in decreased GPX activity only in Ets-1 overexpression cell lines (n = 3). **C)** The protein expression of factors involved in redox regulation was measured via Western blot, including Ets-1, HIF-1α, xCT, GPX-1, and GPX-2, with actin as a loading control (n = 3).

To identify the role of Ets-1 in regulating either of the transsulfuration or Sx_c_^-^ sources of GSH, we have used specific inhibitors of both pathways. Propargylglycine (PPG) irreversibly inhibits the cystathionine γ-lyase enzyme (CGL), thus preventing cysteine synthesis. Sulfasalazine (SAS) is a pharmacological inhibitor of Sx_c_^-^ that prevents the import of cystine from the transporter. Following treatment with inhibitors of the transsulfuration pathway (PPG) or Sx_c_^-^ (SAS), we measured intracellular GSH levels and GPX activity in order to examine the importance of each pathway in cellular redox regulation. Total cellular GSH was decreased by PPG treatment in all cell lines, and SAS did not have any significant effect (Figure 3A). Transsulfuration pathway inhibition with PPG resulted in decreased GPX activity in all cell lines, and Sx_c_^-^ inhibition with SAS caused decreased GPX activity in both non-induced and induced 2008-Ets1 cells (Figure 3B). The protein expression of several factors involved in redox state regulation was examined via Western blot (Figure 3C). Ets-1 is very lowly expressed in 2008 cells compared to 2008-Ets1 cells and their induced counterparts. In addition, the protein levels of xCT, GPX-1, and GPX-2 were increased with overexpression of Ets-1. In 2008 cells, both PPG and SAS treatments caused a decreased in hypoxia inducible factor 1α (HIF-1α) protein. In contrast, PPG treatment resulted in the increase of both GPX-1 and GPX-2 protein levels. In 2008-Ets1 cells, inhibition of the transsulfuration pathway with PPG decreased the protein expression of HIF-1α, xCT, and GPX-1, while SAS decreased the expression of GPX-1. Induced 2008-Ets1 cells showed decreases in HIF-1α, xCT, and GPX-2 protein following PPG treatment, and decreased GPX-2 after treatment with SAS.

### Ets-1 recruits Sx_c_^-^ to maintain glutathione pool under oxidative stress

**Figure 4:**
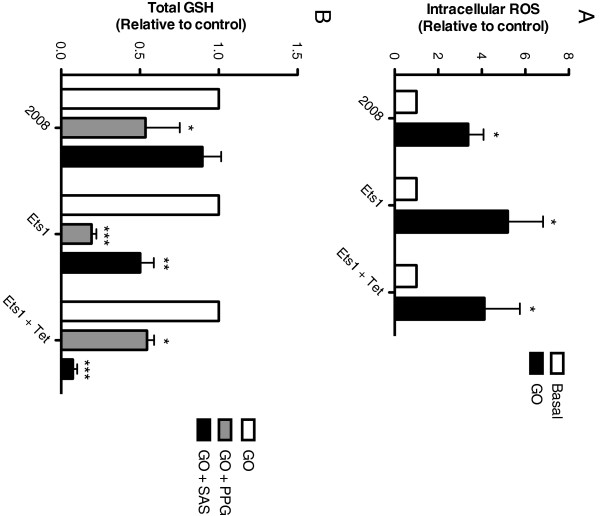
**Ets-1 recruits Sx**_**c**_^**- **^**to maintain glutathione pool under oxidative stress. A)** Glucose oxidase was used to induce oxidative stress in cultures, and successfully increased intracellular ROS levels in all cell lines. **B)** Under oxidative stress, PPG treatment resulted in decreased intracellular GSH levels. The amount of GSH was decreased by SAS in only ovarian cancer cells that express Ets-1 in abundance.

Glucose oxidase treatment was used to induce oxidative stress, resulting in increased intracellular ROS in all cell lines (Figure 4A). Intracellular GSH levels were decreased in response to transsulfuration inhibition in all cell types, but only Ets-1 overexpressing cells displayed decreased GSH levels following inhibition of Sx_c_^-^ (Figure 4B). The decrease in GSH levels following sulfasalazine treatment was inversely correlated with increased Ets-1 expression.

### Ets-1 redox regulation involves changes in HIF-1α and GPX-2 protein levels

**Figure 5:**
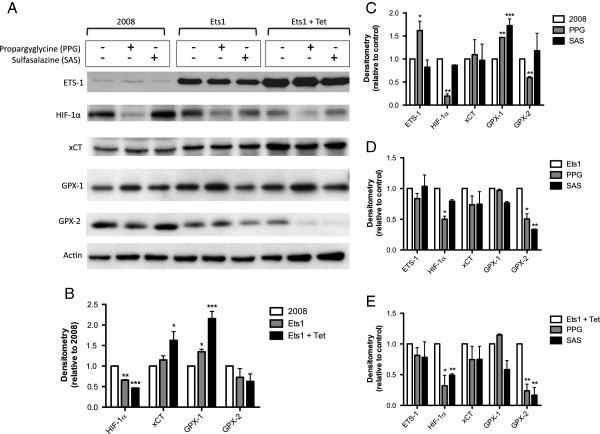
**Ets-1 redox regulation involves changes in HIF-1 and GPX-2 protein levels. A)** The protein expression of redox balance-related factors was examined by Western blot. **B)** Both xCT and GPX-1 protein are increased in correlation with Ets-1 overexpression, while HIF-1α and GPX-2 showed a trend to decreased with higher levels of Ets-1. **C)** In 2008 cells, HIF-1α and GPX-2 were decreased in response to PPG treatment, while GPX-1 was increased with both PPG and SAS. **D)** In 2008-Ets1 cells, decreased protein expression was observed for HIF-1α and GPX-2, while SAS also decreased GPX-2 protein levels. **E)** Similarly, tetracycline-induced 2008-Ets1 cells displayed decreased protein expression of HIF-1α and GPX-2 following PPG and SAS treatment.

Following the induction of oxidative stress, the protein expression of the redox-related proteins HIF-1α, xCT, GPX-1, and GPX-2 were determined via Western blot (Figure 5A). When comparing 2008, 2008-Ets1, and induced 2008-Ets1 cells, the expression of HIF-1α is decreased, while the expression of xCT and GPX-1 are increased in response to Ets-1 overexpression (Figure 5B). In response to inhibitor treatment, PPG decreased HIF-1α and GPX-2 expression in all cell lines, in addition to increasing Ets-1 and GPX-1 protein levels in 2008 cells (Figure 5C-D). SAS treatment resulted in similar observations in Ets-1 overexpressing cells where inhibition of Sx_c_^-^ decreased GPX-2 in both Ets-1-expressing lines, and also decreased HIF-1α protein levels in induced 2008-Ets1 cells (Figure 5C-D).

## Discussion

In the present study, we have identified Ets-1 as a mediator of glutathione antioxidant capacity in an ovarian cancer model of Ets-1 overexpression, shown that Ets-1 recruits the membrane antiporter Sx_c_^-^ to increase intracellular GSH levels, and described a potential mechanism to overcome drug resistance in ovarian cancer cells. The importance of Ets-1 in cancer has been investigated extensively with regards extracellular matrix remodeling and angiogenesis [19,20]. Our laboratory recently identified Ets-1 as a key regulator of cancer metabolism that encourages glycolytic dependence and decreases oxidative phosphorylation [10]. To our knowledge, the present study is the first report to define a role for Ets-1 in redox state regulation of intracellular glutathione levels in cancer cells.

**Figure 6:**
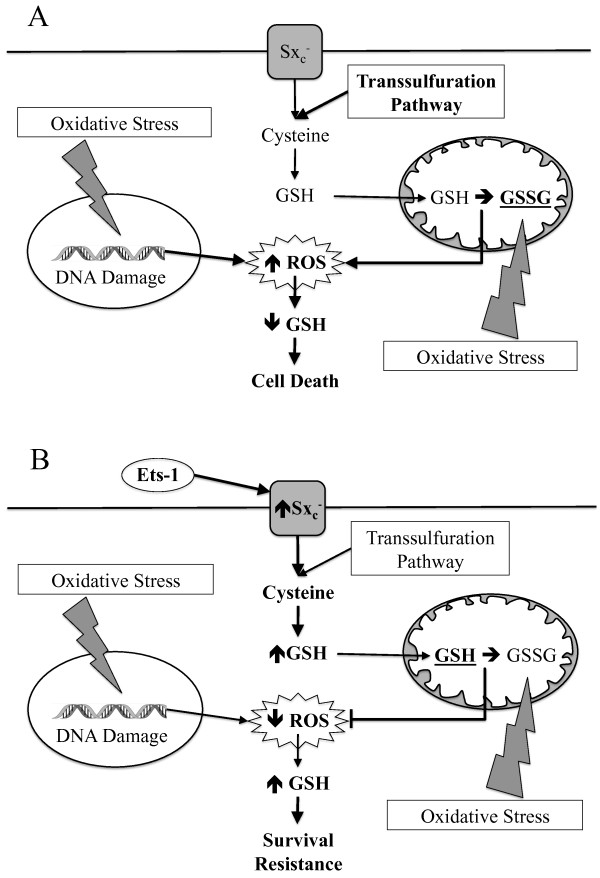
**Proposed mechanism for Ets-1 mediated drug resistance in ovarian cancer. A)** In the absence of abundant Ets-1 expression, the transsulfuration pathway is the main cysteine source for glutathione synthesis. Treatment with chemotherapeutic agents induces oxidative stress that causes DNA damage and GSH depletion by increasing intracellular ROS, leading to cell death. **B)** Under the control of Ets-1 expression, Sx_c_^-^ activity is increased to bolster cysteine stores thereby increasing intracellular glutathione. High levels of glutathione prevent oxidative stress-inducing therapies from causing an accumulation of ROS. Thus, Ets-1 overexpression may play an important role in the drug resistance often observed in aggressive ovarian cancer.

Cellular redox balance is an important regulator of cancer cell proliferation, apoptotic evasion, and therapeutic resistance. Thus, Ets-1-mediated alterations in glutathione antioxidant activity likely account, at least in part, for the associations observed with overexpression of this factor and poor prognosis, advanced malignancy, and enhanced metastatic potential in several types of cancer [21-30]. We have chosen to further characterize the function of Ets-1 in ovarian cancer based on our previous work using the 2008/C13* ovarian cancer model of cisplatin resistance [8]. C13* cells are a variant of 2008 adenocarcinoma cells that were generated from 2008 cells subjected to 13 consecutive rounds of cisplatin treatment. These cells display elevated mitochondrial membrane potential, enhanced DNA repair mechanisms, and altered transcription factor expression, which all contribute to therapeutic resistance [8]. As Ets-1 is prominently overexpressed in C13* cells, we generated a stable overexpression model of Ets-1 in 2008 cells and have since characterized novel roles for Ets-1 in ovarian cancer cells using this model [8,10,31]. In this study, we have shown that overexpression of Ets-1 leads to decreased intracellular ROS levels, concomitantly with increased intracellular GSH levels and GPX activity. These findings are significant as they suggest a mechanism for the increased tolerance to oxidative stress observed in aggressive, drug-resistant ovarian cancer cells (Figure 6).

GSH plays a key role in the antioxidant capabilities of cancer cells, and high levels confer protection against oxidative stress-inducing chemotherapy and ionizing radiation. The development of targeted agents that deplete GSH would improve the ability of these agents to induce cancer cell death, as low levels of GSH trigger mitochondrial apoptosis, necrosis, and autophagy [14,15,32,33]. Depletion of GSH can be achieved through the inhibition of GSH transport, enhancement of efflux from cells, or by limiting cysteine availability causing impaired GSH synthesis. We have reported here that the overexpression of Ets-1 induces increased expression and activity of Sx_c_^-^, suggesting that GSH depletion by limiting intracellular cysteine might be a viable therapy in these cells. Treatment with SAS, an anti-inflammatory drug that blocks Sx_c_^-^ transport, resulted in a significant decrease in GPX activity but not total GSH levels under basal conditions. However, decreases in GSH levels and GPX activity were achieved following treatment with PPG, which blocks cysteine synthesis by the transsulfuration pathway. Therefore, we suggest that under basal conditions ovarian cancer cells rely predominantly on the transsulfuration pathway to maintain GSH levels irrespectively of Ets-1 expression level.

As many therapeutic agents function by inducing oxidative stress, we also examined the effects of SAS and PPG on ovarian cancer cells exposed to such conditions by glucose oxidase treatment. Interestingly, we have shown that GSH depletion following SAS treatment correlates with Ets-1 overexpression levels, where cancer cells that express high levels of Ets-1 display greatly decreased GSH levels following Sx_c_^-^ blockade. Protein expression of the Sx_c_^-^ catalytic subunit xCT and glutathione peroxidase enzyme GPX-1 were increased in correlation with increasing Ets-1 expression suggesting that they may be target genes of Ets-1. Interestingly, the protein expression of HIF-1α and GPX-2 were decreased concomitantly in response to transsulfuration pathway inhibition in all cell lines, an effect that was mimicked with SAS treatment only in Ets-1 overexpression lines. These findings not only suggest that HIF-1α and GPX-2 are involved in the maintenance of intracellular GSH levels, but also that cellular redox state can influence the expression, and likely the stability of HIF-1α.

The master regulator of cellular redox state is arguably the transcription factor nuclear factor (erythroid-derived)-like-2 (Nrf2), which initiates the antioxidant response pathway primarily responsible for cellular defense against oxidative stress and is frequently mutated in cancer [34]. Our laboratory has previously established that Ets-1 is a target gene of Nrf2 via binding of an antioxidant response element within the Ets-1 promoter region [31]. However, a hypoxia response element within the promoter region was also found to be involved in the transcriptional induction of Ets-1 expression, suggesting an important role for HIF-1α. The impact of redox balance fluctuation on HIF-1α expression and stability is well defined, thus we examined HIF-1α protein expression in our ovarian cancer model following GSH depletion. Not surprisingly, the expression of HIF-1α decreased in correlation with decreased ROS levels in our 2008 cell model and following transsulfuration pathway blockade. Under oxidative stress, HIF-1α was decreased in response to SAS treatment only in cells that overexpress Ets-1 suggesting that GSH depletion regulates HIF-1α levels in ovarian cancer cells. In agreement with our findings, the ability of redox state fluctuations to affect HIF-1α expression was recently observed in hepatocellular carcinoma cells following inhibition of GSH synthesis by the chemotherapeutic agent buthionine sulphoximine [35], as well as in astrocytes where GSH depletion decreased HIF-1α expression [36]. Considering these results, further investigation into the interrelationship between Ets-1, Nrf2, and HIF-1α in ovarian cancer cells is warranted.

Our results suggest that agents that deplete GSH levels may be effective sensitizing agents in aggressive, drug-resistant ovarian cancers when used as a pre-treatment prior to conventional oxidative stress-inducing therapies. There is precedence for such a therapeutic strategy, as SAS specifically has shown therapeutic promise in pancreatic [18,37], lung [38,39], hepatocellular [40], prostate [41], and breast cancers [42]. These studies illustrated that SAS has the ability to decrease tumour growth *in vivo* in several types of cancer, and can also enhance the efficacy of the chemotherapeutic agents etoposide [37], gemcitabine [18,37], and doxorubicin [39,42]. Gemcitabine and cisplatin are commonly used in combination together, and since Ets-1 is involved in cisplatin resistance, further study of the validity of pre-treatment of GSH-depleting agents with gemcitabine and cisplatin combination therapy for ovarian cancer is warranted [8]. The diverse functional roles of Ets-1 in a variety of cancer types truly illustrate the potential use of Ets-1 inhibition as an effective therapeutic target, although the limitations to such an approach are significant. Several other Ets factors share sequence homology with Ets-1, and thus direct inhibition of Ets-1 specifically is unreasonable. Therefore, further examination of the regulation of Ets-1 and the functional consequences of its overexpression are of particular interest to the development of novel therapeutic approaches for ovarian cancer.

## Conclusions

Ovarian cancer cells that overexpress Ets-1 display a more active glutathione antioxidant system, a characteristic that could account for the chemotherapeutic resistance previously observed in these cells [8]. Under conditions of oxidative stress, Ets-1 recruits the Sx_c_^-^ transporter to bolster cellular glutathione levels resulting in decreased ROS levels, increased intracellular GSH, and increased GPX activity. In blocking the activity of Sx_c_^-^, GSH levels are depleted and cell viability is compromised, suggesting that this approach may reduce resistance to oxidative stress-inducing therapies. Although we have focused on an ovarian cancer cell model for this study, the potential applicability of these findings is broad because Ets-1 overexpression is frequently associated with a wide spectrum of aggressive, advanced cancers.

## Methods

### Cell culture and treatments

The human ovarian carcinoma cell line 2008 was kindly provided by Dr. Paul Andrews (Georgetown University, Rockville, MD, United States) [43], and were maintained in RPMI 1640 medium supplemented with 10% fetal bovine serum and 2% penicillin/streptomycin. Stable cell line 2008-Ets1 was maintained in growth medium as described with the addition of 200 ng/ml selective antibiotic (zeocin). All cells were kept at 37°C in a humidified atmosphere of 5% CO_2_. All the experiments performed with these cell lines were approved by the Presidential Biosafety Advisory Committee of McMaster University, Canada. Media and supplements were purchased from Invitrogen Life Technologies (ONT, Canada). When cells reached 40-50% confluency, the cultures were exposed to either PPG or SAS at 2.5 mM and 50 uM respectively for 24 hours prior to isolation or analysis. To induce oxidative stress in cultures, cells were treated with 10 mU/mL glucose oxidase and/or PPG/SAS for 24 hours prior to isolation or analysis.

### Western Blotting and densitometry analysis

Whole cell lysates were collected, and 30 μg of protein was separated by 10% SDS-PAGE electrophoresis, transferred to PVDF membrane, and blocked for 1 hr in 5% skim milk TBS-T. Membranes were incubated overnight with antibody reactive to Ets-1 (Abcam, MA, United States), HIF-1α (Cell Signaling, MA, United States), xCT (Abcam), GPX1 (Abcam), GPX2 (Abcam), or Actin (Cell Signaling) in 0.5% TBS-T. Following primary antibody incubation, membranes were washed and incubated for 2 hrs with horseradish peroxidase-linked anti-mouse or anti-rabbit IgG secondary antibody as appropriate (Cell Signaling). Proteins were detected by ECL chemiluminescence reagent (Amersham, NJ, United States), and exposed to film. Densitometry analysis was performed using ImageJ software available at http://rsb.info.nih.gov/ij and developed by Wayne Rasband, National Institutes of Health, Bethesda, MD, USA.

### Glutamate release and cell viability assays

Glutamate levels in the culture medium were evaluated using the AMPLEX red® glutamic acid assay kit, which was optimized for higher glutamate concentrations by the omission of L-alanine and L-glutamate pyruvate transaminase from the reaction [44]. Cell viability was measured using Crystal Violet staining with all cell lines seeded at 10,000 cells per well, and normalized to a standard curve of cell numbers.

### Intracellular ROS assay

Intracellular ROS levels were measured using CM_2_-H_2_DCFDA reagent (Invitrogen), which is cleaved once inside the cell allowing the DCF dye to bind to ROS species resulting in fluorescence. Cells were plated in 96-well plates and grown to 70-90% confluency in phenol red-free medium. CM_2_-H_2_DCFDA reagent was reconstituted in DMSO, and 10 μM was added to each experimental well using phenol red-free medium containing 10% FBS. Following a 30 min incubation to allow the dye to load into cells, plates were washed twice with PBS, and allowed to recover in phenol red-free medium for 10 minutes. Plates were then treated with 250 μM H_2_O_2_ and read in a Cytofluor fluorescent plate reader at 485 nm excitation and 530 nm emission for 1 hour. Plates were then stained with Crystal Violet, dried overnight, solubilized with SDS, and read at 570 nm. Arbitrary fluorescent values were normalized to Crystal Violet absorbance values, and reported as Arbitrary Fluorescent Units (AFU).

### Determination of intracellular GSH concentration

Metabolite cell extracts were prepared as described by Rahman *et al.*[45]. Briefly, cultures were grown to 70-80% confluency, washed with PBS, and pelleted twice. Extracts were resuspended in cold extraction buffer containing 0.06% sulfosalicylic acid, homogenized using a 26.5 gauge needle, lyzed via freeze/thaw at −70°C, pelleted, and the supernatant was retained as used for GSH measurement. Intracellular GSH content of the metabolite extracts was measured as described [45]. The concentration of total GSH was normalized to the protein concentration of each extract.

### GPX activity assay

Glutathione peroxidase enzyme activity was measured using the glutathione peroxidase activity kit from Enzo Life Sciences (NY, USA) according to the manufacturer instructions. The GPX activity was normalized to the protein concentration of the each cell extract.

### Statistical analysis

Data is presented as the mean +/− standard deviation from at least three independent experiments. Statistically significant differences between sample groups were determined using a Student’s t-test or ANOVA where applicable, with a p-value ≤ 0.05 considered to be statistically significant (p ≤ 0.05 = *, p ≤ 0.01 = **, p ≤ 0.001 = ***).

## Abbreviations

AFU: Arbitrary fluorescent units; CGL: Cystathionine γ-lyase enzyme; Ets-1: V-ets erythroblastosis virus E26 oncogene homolog 1; GPX: Glutathione peroxidase; GSH: Reduced glutathione; HIF-1α: Hypoxia inducible factor 1α; Nrf2: Nuclear factor (erythroid-derived)-like-2; PPG: Propargylglycine; ROS: Reactive oxygen species; SAS: Sulfasalazine; Sx_c_^-^: System x_c_^-^.

## Competing interests

The authors declare that they have no competing interests.

## Authors’ contributions

MV carried out all experiments, analysis, interpretation of the data, and drafted the manuscript. GS contributed substantially to the design of the study, and revised the manuscript. Both authors have read and approved the final manuscript.
